# Supplementary material for: Blocking the GITR-GITRL pathway to overcome resistance to therapy in sarcomatoid malignant pleural mesothelioma
Source: Commun Biol. 2021 Jul 26;4:914. doi: 10.1038/s42003-021-02430-5 (PMC8313521; doi:10.1038/s42003-021-02430-5)
Supplement: Supplementary file 1 — Description of Supplementary Files [file 42003_2021_2430_MOESM1_ESM.pdf]

Description of Additional Supplementary Files

File Name: Supplementary Data 1

Description: Dataset source for figures 1b, 1d, 2a, 4b, 4d, 5c, 6a, 6b, 6c, 6d, 6e (Excel file).

File Name: Supplementary Data 2

Description: Microarray data deposited in ArrayExpress.
