# Supplementary material for: Blocking the GITR-GITRL pathway to overcome resistance to therapy in sarcomatoid malignant pleural mesothelioma
Source: Commun Biol. 2021 Jul 26;4:914. doi: 10.1038/s42003-021-02430-5 (PMC8313521; doi:10.1038/s42003-021-02430-5)
Supplement: Supplementary file 2 — Supplementary Information [file 42003_2021_2430_MOESM2_ESM.pdf]

Supplementary Materials:

Materials and Methods

Cell viability assay

Human mesothelioma CRL5946 cells (American Type Culture Collection, ATCC) were detached and suspended in RPMI 1640 medium (Gibco, Thermo Fisher Scientific) and exposed to cisplatin in different concentrations at 0.5, 1, 2, and 4 μg/mL overnight or γ-ray radiation (Atomic Energy of Canada, Ltd, Ottawa, Canada) in different dosage at 2.5, 5. 7.5, and 7.5 Gy and then seeded onto 6-well culture plates. We replaced the culture medium 24 hours later. For short-term cytotoxicity determination, we initially loaded 10^5 cells onto each well. After 4 days of incubation, we washed twice with warm PBS and resuspended the attached cells in medium. Then the number of viable cells were counted by Cell Counter (Vi-CELL XR, BECKMAN COULTER).

Real-time reverse transcription PCR analysis

Human mesothelioma CRL5946 cells were exposed to 2 μg/mL cisplatin or 7.5 Gy Cs-137 irradiation for indicated time points. Total RNA was extracted from cells by using QIAzol Lysis Reagent (QIAGEN) and RNeasy Microarray Tissue Mini Kit (QIAGEN). cDNA was synthesized with High Capacity cDNA Reverse Transcription Kit (ThermoFisher Scientific) on a C1000 Touch ™ Thermal Cycler (BIORAD) following manufacturer's protocols. Regular PCR was done to establish reverse transcription PCR (RT-PCR) standards of all targets genes including GITRL, GITR, and housekeeping gene HPRT1. DNA fragments were obtained from regular PCR on a C1000 Touch ™ Thermal Cycler (Bio-Rad). A probe-based real-time PCR approaches for quantitative measurement of targets genes was carried out on the CFX384 Touch real-time PCR detection system (BIO-RAD). PCR composed of 20X PrimePCR Probe Assay, 2X SsoAdvanced™ Universal Supermix, 2ul 25ng/ul cDNA X 45 cycles. The Probes of PrimePCR Probe Assay of house-keeping gene and all target genes were purchased from BIO-RAD

Laboratories, Inc.

Flow cytometry analysis

Cultured CRL5946 cells were detached from culture plate and resuspended in EasySep buffer (STEMCELL Tech.). The sequential sorting of GITR-GITRL+, GITR+GITRL- and GITR-GITRL- was done by using human-specific antibodies: GITRL-PE conjugated (Clone#109101, R&D Systems Co.) and GITR-PE conjugated (Clone#621, BioLegend Inc.) and EasySep™ Release Human PE Positive Selection Kit following the manufacturer's instructions. Sorted cells were culture in RPMI 1640 medium for 0, 7, and 16 days, separately. For staining surface GITR and GITRL, Cells were stained for 10 minutes at 4 ^{o}C with a CD16/CD32 Fc block (BioLegend Inc.) and then a combination of the following human-specific antibodies: GITRL-APC conjugated (Clone#109101, R&D Systems Co.) and GITR-PE conjugated (Clone#621, BioLegend Inc.). All samples were then washed twice with FACS buffer and fixed with 2% Paraformaldehyde for at least 30 minutes. BD LSR II flow cytometer (BD Biosciences) and FlowJo V10 software (FlowJo LLC) was then used to analyze the expression of GITR and GITRL on MPM cells.

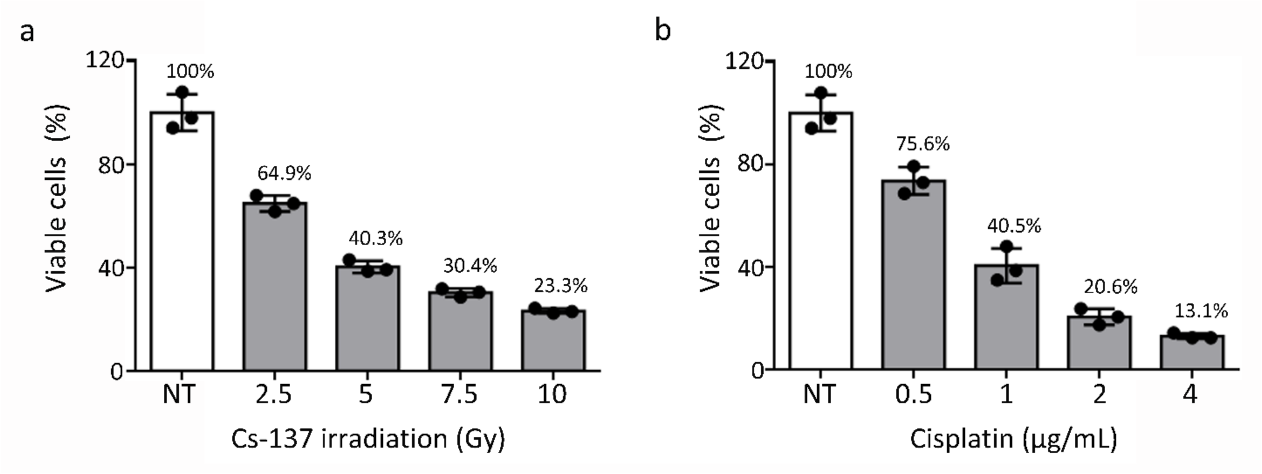

Fig. S1. Dosage-dependent cytotoxicity of Cisplatin and Cs-137 irradiation in CRL5946 MPM cells. Representative figure showed the relative number of viable cells of CRL5946 MPM cells 96 hours after different dosage of Cs-137 irradiation **(A)** and cisplatin exposure **(B)**. NT: No Treatment.

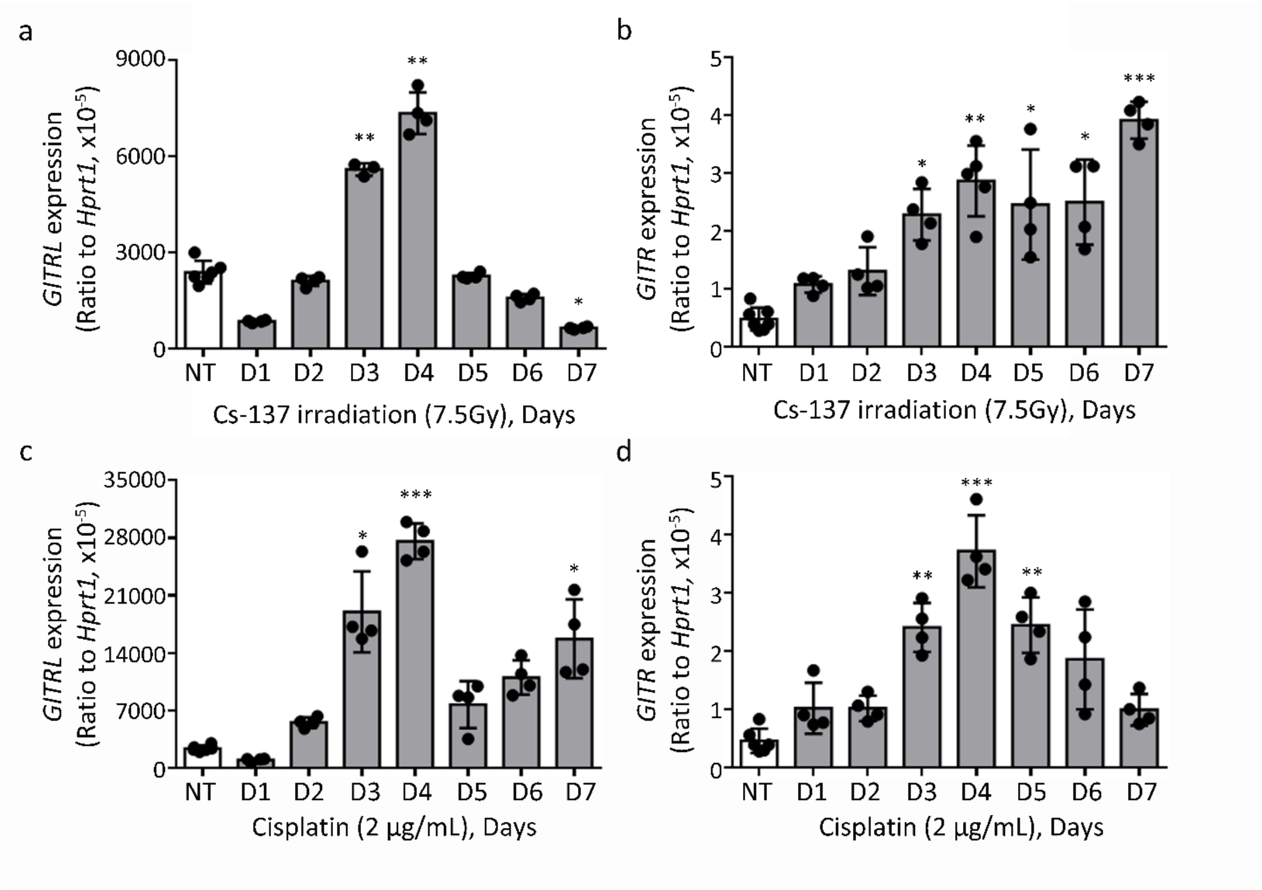

Fig. S2. Increased of GITRL and GITR expression in CRL5946 treated with Cs-137 or cisplatin in a time-depend manner. Representative figures showed the change of GITR and GITRL expression in CRL5946 cells by time after 7.5 Gy Cs-137 irradiation (**A** and **B**) or 2ug/ml cisplatin treatment (**C** and **D**). The results of qRT-PCR showed the expression level of GITR and GITRL relative to house-keeping gene, Hprt1. NT: No Treatment. *P<0.05; **P<0.01; ***P<0.001 determined by Mann-Whitney U test.

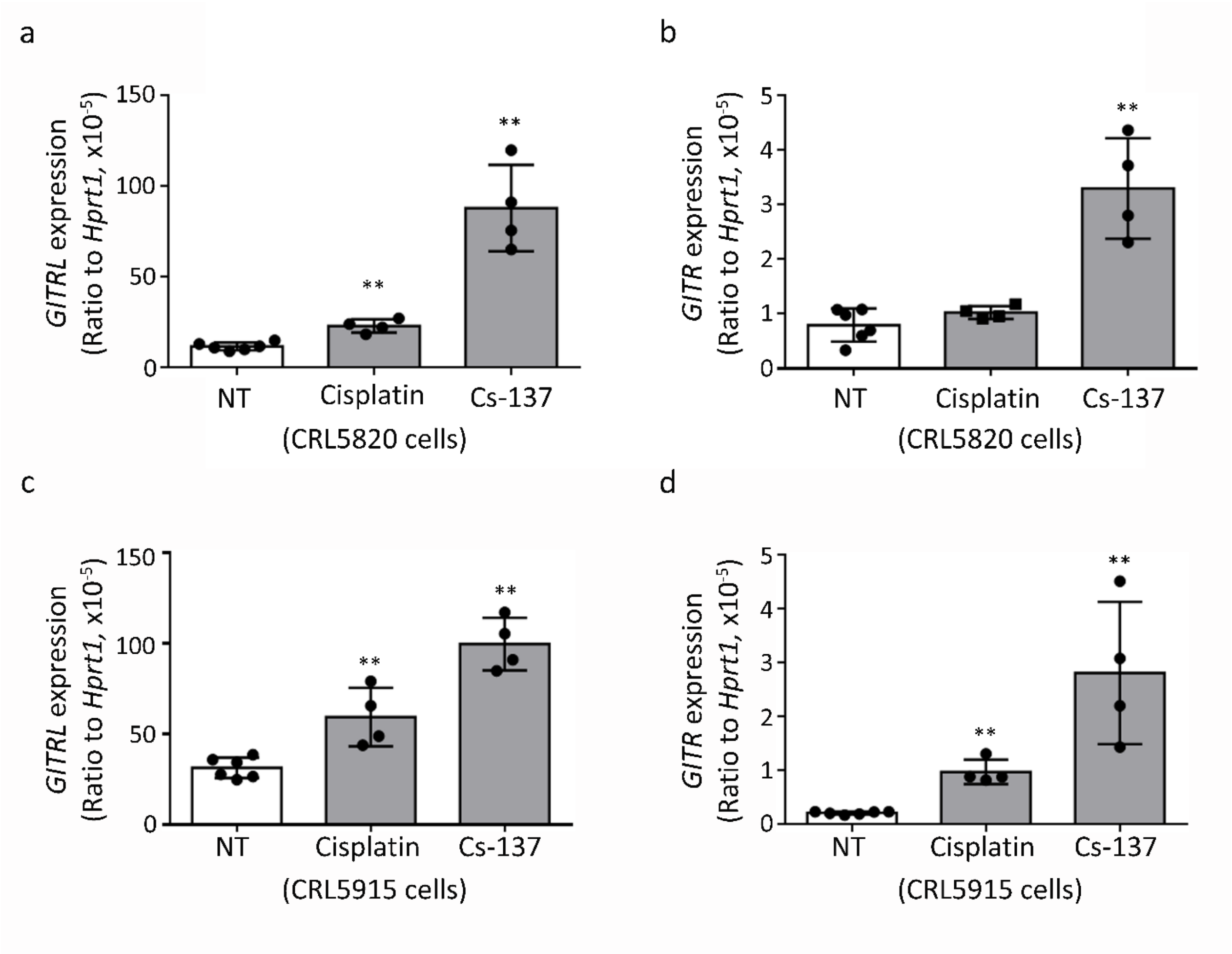

Fig. S3. Increased of GITR and GITRL expression in CRL5820 and CRL5915 treated with Cs-137 or cisplatin. CRL5820 or CRL5946 cells were exposed to 2 ug/mL cisplatin for 24 hours or 7.5Gy Cs-137 irradiation. The mRNA was collected 4 days later for qRT-PCR analysis of GITR and GITRL. The expression of GITR and GITRL were increased in CRL5820 (**A** and **B**) and CRL5915 (**C** and **D**) cells, compared with non-treated (NT). ***P*<0.01 determined by Mann-Whitney U test.

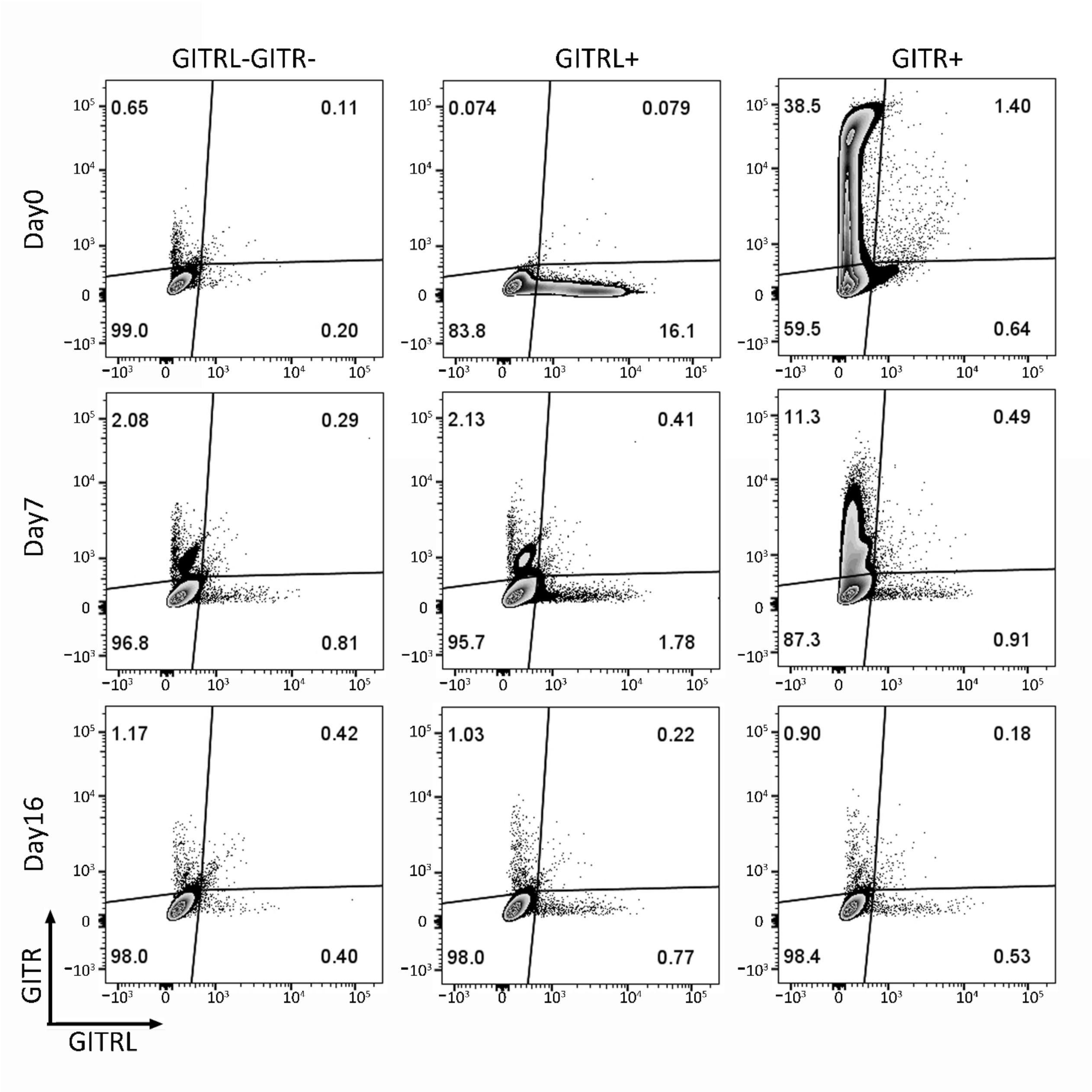

Fig. S4. The stochastic interconversion of phenotype contributes to heterogeneity of CRL5946 cells. The GITR+, GITRL+ and GITR-GITRL- cells of CRL5946 cells were sequentially sorted by using magnetic beads methods. After sorting, the GITR+ sorted group can reach up to 38.5%, the GITRL+ sorted group can reach up to 16.1 % (upper panel). After cultivating on plate for 7 days, the distribution of GITR+ and GITRL+ cells gradually went back to normal distribution (middle panel). After cultivating on plate for 16 days, the distribution of GITR+ and GITRL+ was totally back to normal equilibria among the three initially sorted subpopulations.

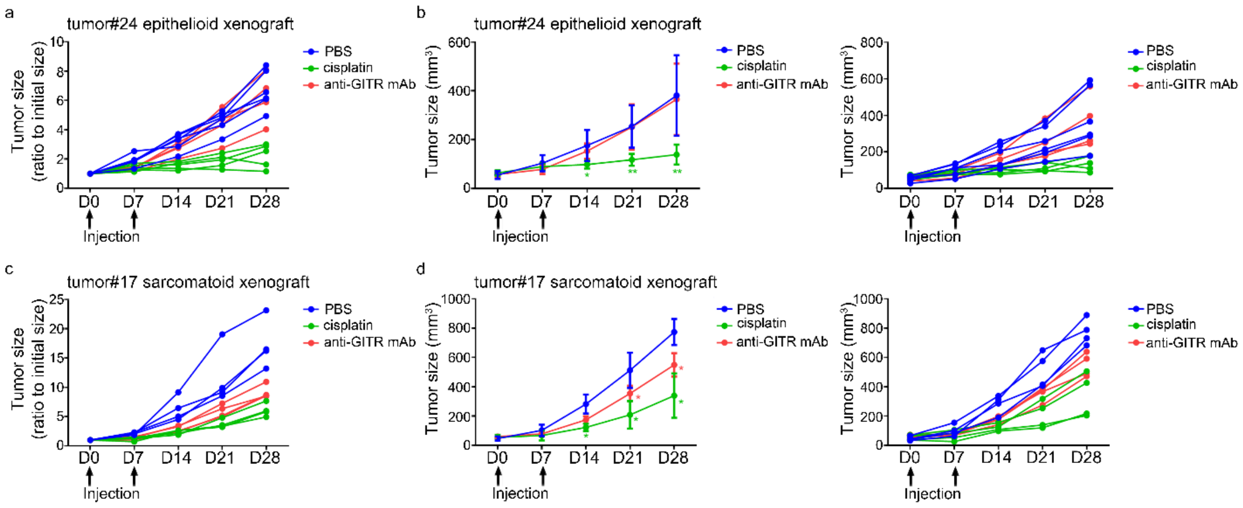

Fig. S5. The individual tumor growth is presented in a ratio to initial size (normalized) for the tumor#24 epithelioid xenograft (A) and tumor#17 sarcomatoid xenograft (C). The average tumor size (left panel) as well as the individual figure size (right panel) for each mouse is then presented for the epithelioid xenograft (B) and for the sarcomatoid xenograft (D).

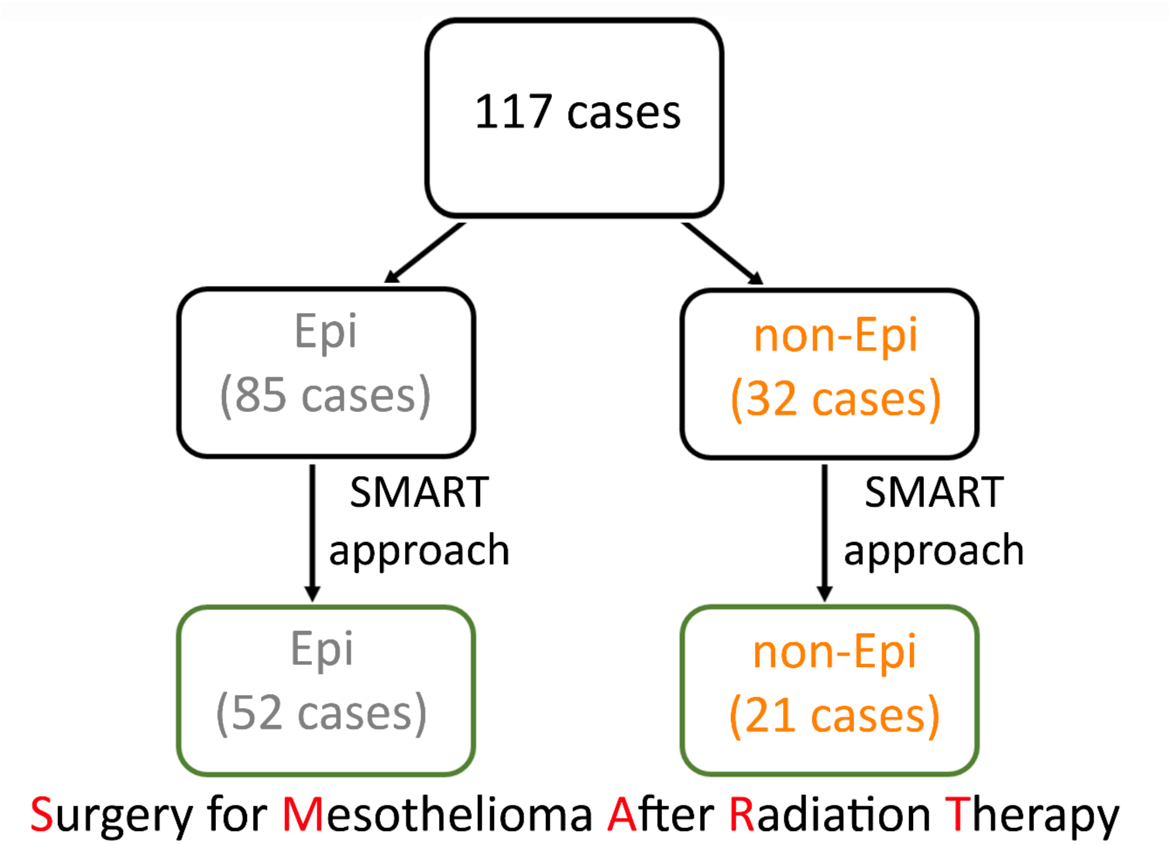

Fig. S6. Demographics of MPM patients who were evaluated in our institution from November 2003 to October 2016. A total of 117 MPM cases were assessed with pathology blocks available for immunostaining. There were 85 cases of epithelioid subtype and 32 cases of non-epithelioid subtype histologically. In total, 73 cases including 52 epithelioid subtype and 21 non-epithelioid subtype received surgery for mesothelioma after radiation therapy (SMART).

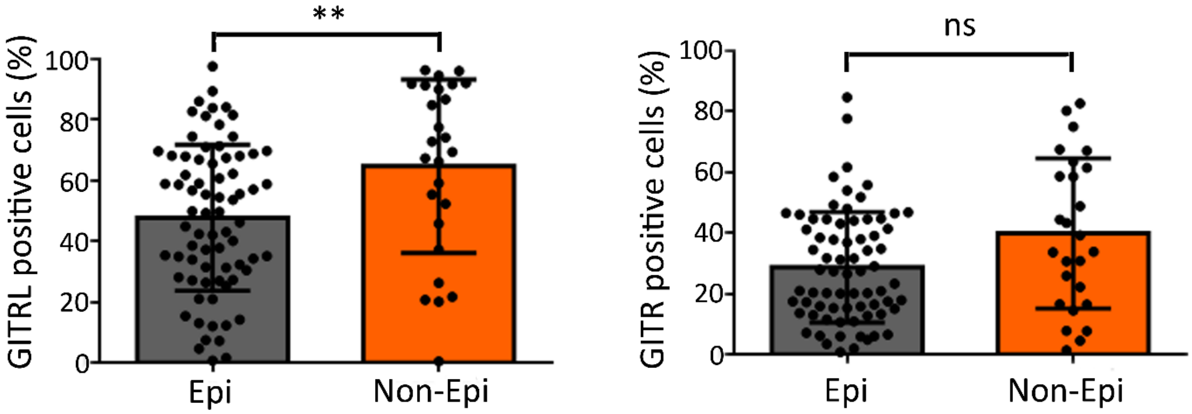

Fig. S7. Immunochemistry staining showed that the percentage of GITRL and GITR positive cells were higher in non-epithelioid subtype than in epithelioid subtype. Immunohistochemistry results were quantified by Definiens Tissuestudio 4.0 software. ** p = 0.01, determined by Mann-Whitney U test.

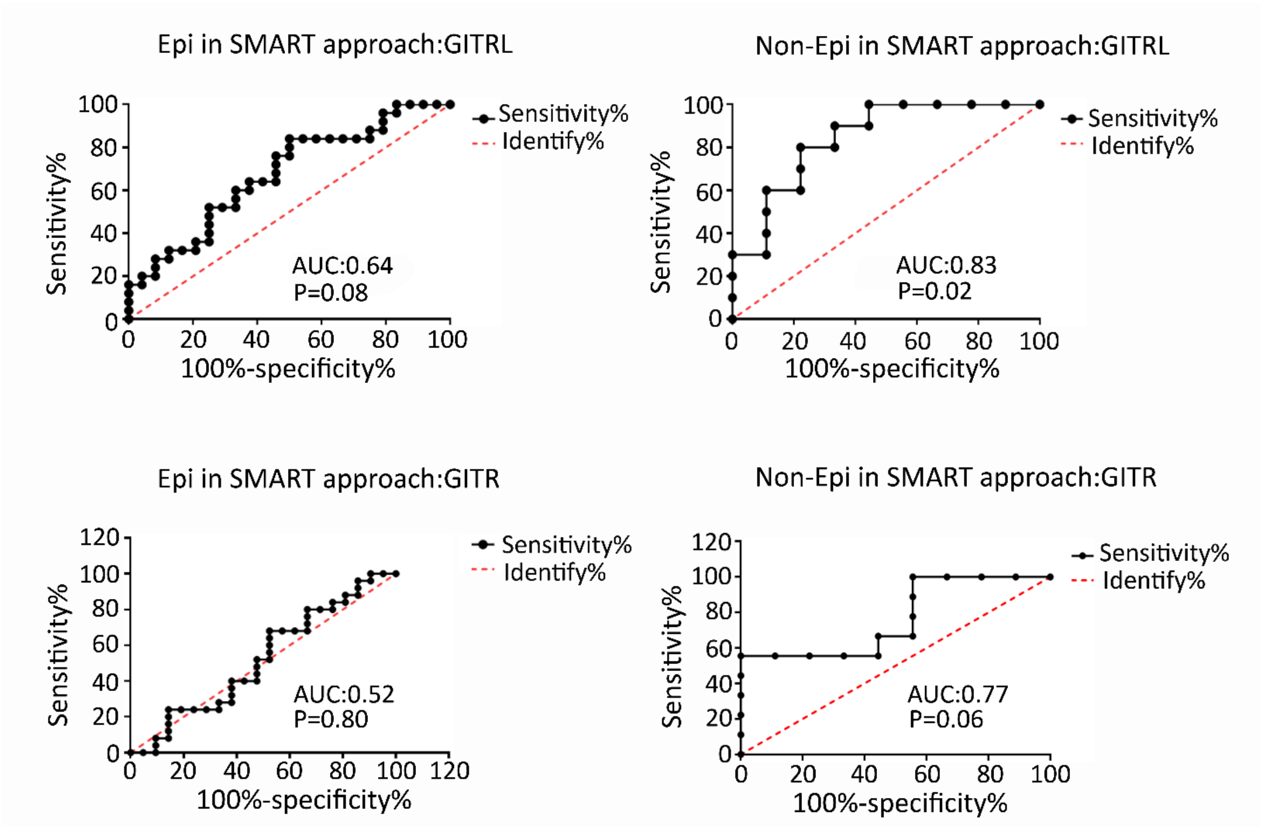

Fig. S8. ROC curve based on the average intensity of GITR and GITRL for predicting better or worse survivals than the median survival in patients with Epi. and non-Epi. subtype after SMART approach.

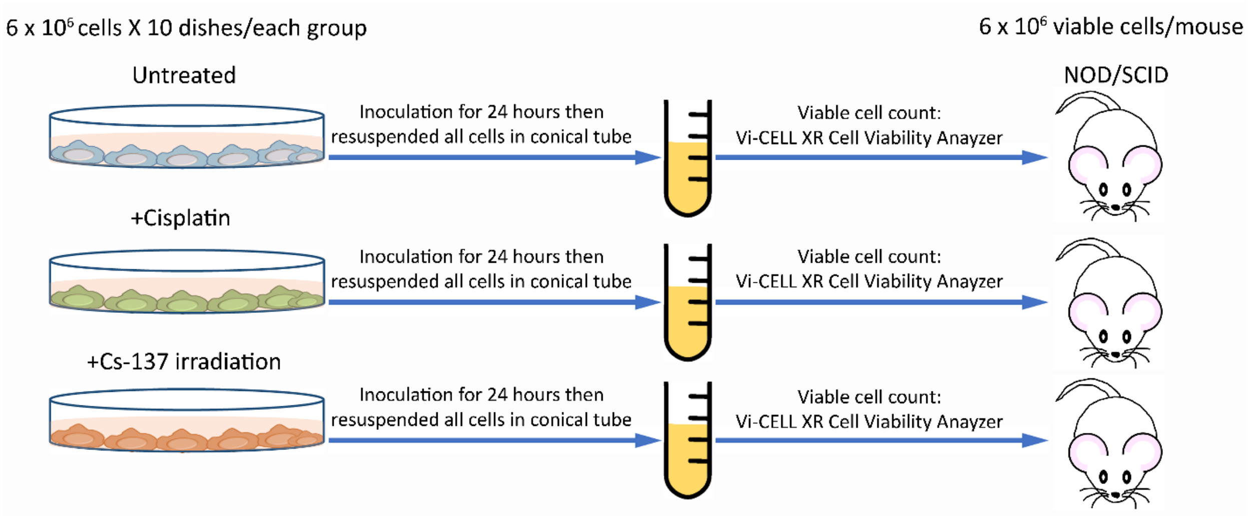

Fig. S9. Schematic representation of the in vivo experiments.

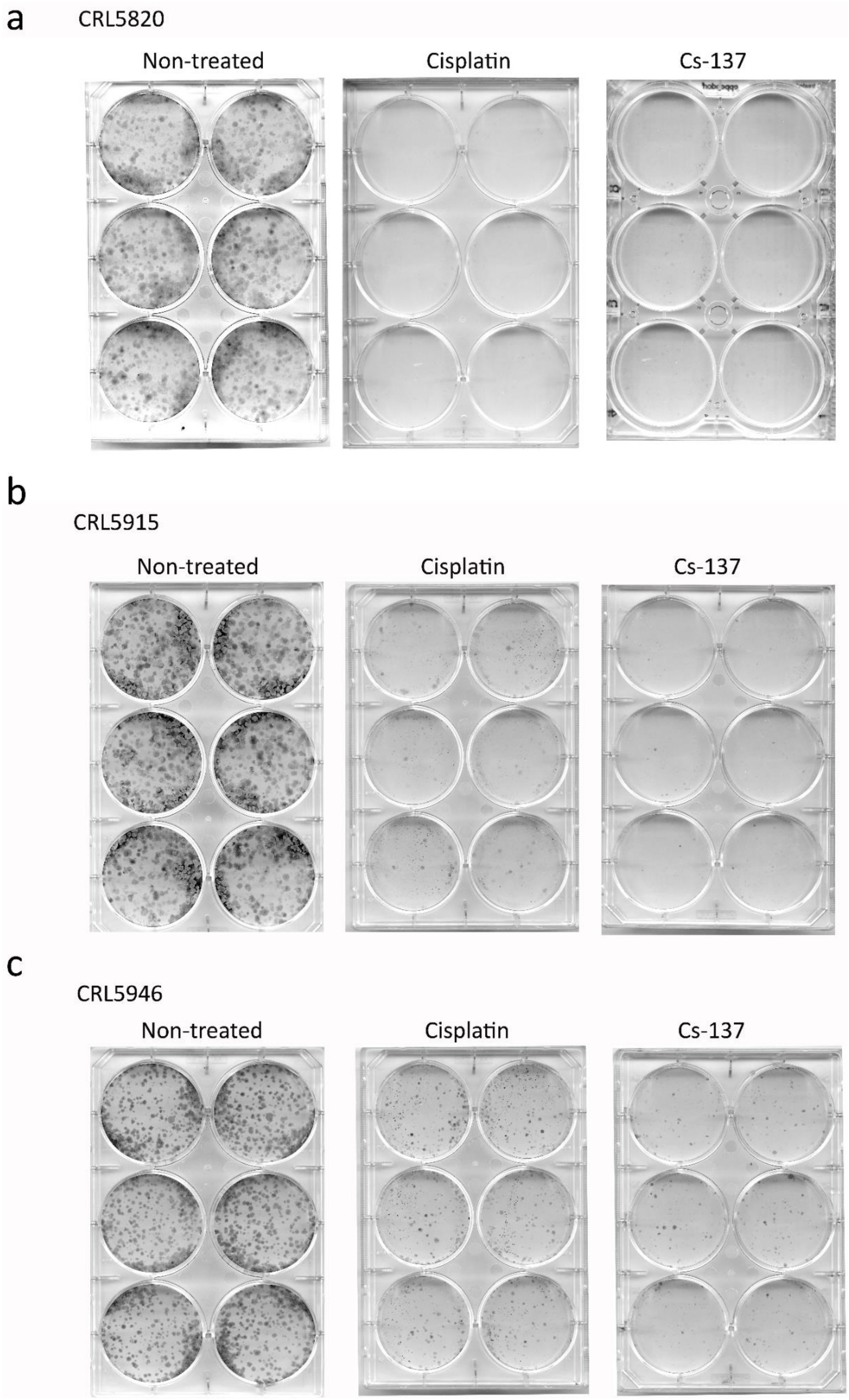

Fig. S10. Original results shown in Figure 1E.

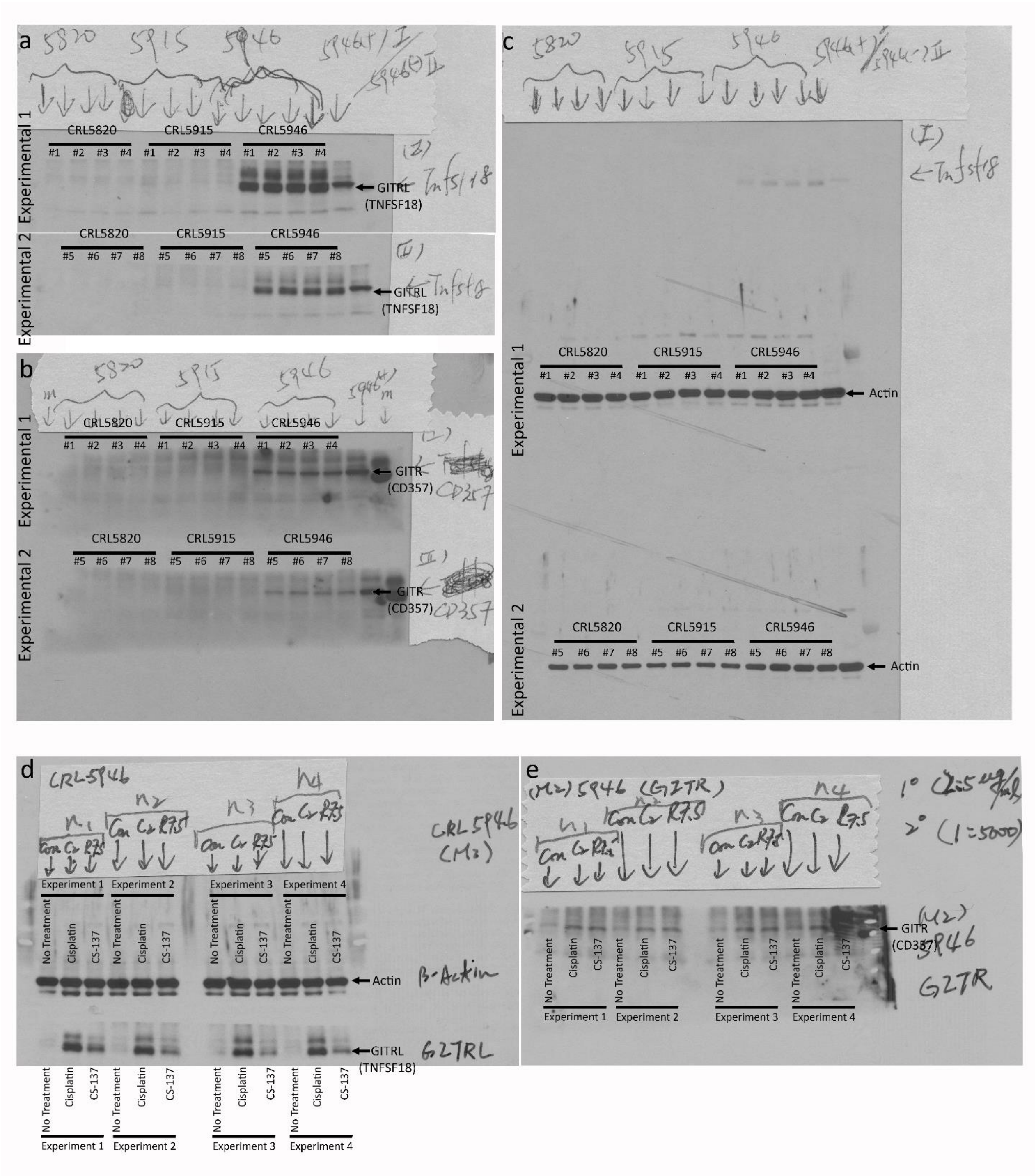

Fig.S11. Supplementary of unprocessed western blot data. (A, B, and C) Unprocessed western blot results of GITRL, GITR and Actin respectively in Fig 1C. (D and E) Unprocessed western blot results of GITRL, Actin, and GITR represented in Fig 2B.
