# Supplementary material for: Blocking the GITR-GITRL pathway to overcome resistance to therapy in sarcomatoid malignant pleural mesothelioma
Source: Commun Biol. 2021 Jul 26;4:914. doi: 10.1038/s42003-021-02430-5 (PMC8313521; doi:10.1038/s42003-021-02430-5)
Supplement: Supplementary file 4 — Reporting Summary [file 42003_2021_2430_MOESM4_ESM.pdf]

nature research

Corresponding author(s): Marc de Perrot

Last updated by author(s): Jun 5, 2021

Reporting Summary

Nature Research wishes to improve the reproducibility of the work that we publish. This form provides structure for consistency and transparency in reporting. For further information on Nature Research policies, see our Editorial Policies and the Editorial Policy Checklist.

Statistics

For all statistical analyses, confirm that the following items are present in the figure legend, table legend, main text, or Methods section.

n/a	Confirmed	
☐	☒	The exact sample size (*n*) for each experimental group/condition, given as a discrete number and unit of measurement
☒	☐	A statement on whether measurements were taken from distinct samples or whether the same sample was measured repeatedly
☒	☐	The statistical test(s) used AND whether they are one- or two-sided<br>*Only common tests should be described solely by name; describe more complex techniques in the Methods section.*
☒	☐	A description of all covariates tested
☒	☐	A description of any assumptions or corrections, such as tests of normality and adjustment for multiple comparisons
☒	☐	A full description of the statistical parameters including central tendency (e.g. means) or other basic estimates (e.g. regression coefficient) AND variation (e.g. standard deviation) or associated estimates of uncertainty (e.g. confidence intervals)
☒	☐	For null hypothesis testing, the test statistic (e.g. *F*, *t*, *r*) with confidence intervals, effect sizes, degrees of freedom and *P* value noted<br>*Give P values as exact values whenever suitable.*
☒	☐	For Bayesian analysis, information on the choice of priors and Markov chain Monte Carlo settings
☒	☐	For hierarchical and complex designs, identification of the appropriate level for tests and full reporting of outcomes
☒	☐	Estimates of effect sizes (e.g. Cohen's *d*, Pearson's *r*), indicating how they were calculated

Our web collection on statistics for biologists contains articles on many of the points above.

Software and code

Policy information about availability of computer code

Data collection	No software was used.
Data analysis	Graphpad Prism 6, FlowJO V10 software, and Bio-Rad CFX manger V3.1 software

For manuscripts utilizing custom algorithms or software that are central to the research but not yet described in published literature, software must be made available to editors and reviewers. We strongly encourage code deposition in a community repository (e.g. GitHub). See the Nature Research guidelines for submitting code & software for further information.

Data

Policy information about availability of data

All manuscripts must include a data availability statement. This statement should provide the following information, where applicable:

- Accession codes, unique identifiers, or web links for publicly available datasets
- A list of figures that have associated raw data
- A description of any restrictions on data availability

The authors confirm that the data supporting the findings of this study are available within the article and its supplementary materials. The clinical results are not publicly available due to ethical restrictions. The clinical findings of this study as well as all other data are available from the corresponding author on reasonable request.

Field-specific reporting

Please select the one below that is the best fit for your research. If you are not sure, read the appropriate sections before making your selection.

☒ Life sciences ☐ Behavioural & social sciences ☐ Ecological, evolutionary & environmental sciences

For a reference copy of the document with all sections, see nature.com/documents/nr-reporting-summary-flat.pdf

Life sciences study design

All studies must disclose on these points even when the disclosure is negative.

Sample size	The sample size (n) of each experiment is provided in the corresponding figure captions in the main manuscript and supplementary information files. Samples sizes were chosen to support meaningful conclusions. The effect size and standard deviation were taken from similar experiments performed in the authors laboratory.
Data exclusions	No data was excluded from the analyses.
Replication	All in vitro and in vivo experiments were replicated successfully at least 3 times.
Randomization	Mice were randomly allocated to each of the treatment groups.
Blinding	Investigators were blinded to group allocation during the data collection.

Reporting for specific materials, systems and methods

We require information from authors about some types of materials, experimental systems and methods used in many studies. Here, indicate whether each material, system or method listed is relevant to your study. If you are not sure if a list item applies to your research, read the appropriate section before selecting a response.

Materials & experimental systems

n/a	Involved in the study	
☐	☒	Antibodies
☐	☒	Eukaryotic cell lines
☒	☐	Palaeontology and archaeology
☐	☒	Animals and other organisms
☒	☐	Human research participants
☐	☒	Clinical data
☒	☐	Dual use research of concern

Methods

n/a	Involved in the study	
☒	☐	ChIP-seq
☐	☒	Flow cytometry
☒	☐	MRI-based neuroimaging

Antibodies

Antibodies used	rabbit monoclonal anti-human GITRL antibody (Clone:EPR20583, Abcam plc.)<br>mouse monoclonal anti-human GITR antibody (Clone:2H4, SIGMA-ALDRICH Co.)<br>anti- ß -Actin antibody (rabbit anti-human, BioLegend Inc.)<br>horseradish peroxidase-conjugated anti-rabbit IgG (Clone:4064, Biolegend Inc.)<br>anti-mouse IgG antibodies (Clone:4053, Biolegend Inc.)<br>anti-human GITRL antibody (Clone:109101, R&D SYSTEMS)<br>anti-mouse IgG Antibody-AF488 conjugated (Invitrogen)<br>anti-human GITR antibody-PE conjugated (Clone#621, Biolegend Inc.)<br>anti-human GITRL-APC conjugated (Clone#109101, R&D Systems co.)<br>anti-human GITR-PE conjugated (Clone#621, BioLegend Inc.)<br>monoclonal Mouse IgG1 (clone # 11711R, R&D Systems Co.)<br>monoclonal anti-GITR antibody (clone # 110416, R&D Systems Co.)<br>Primary antibodies against GITR (Polyclonal, ThermoFisher Inc.)<br>Primary antibodies GITRL (Polyclonal, ThermoFisher Inc.)
Validation	Antibodies were used according to the manufacturer recommendations.

Eukaryotic cell lines

Policy information about cell lines

Cell line source(s)	Human mesothelioma cell lines, NCI-H28 [H28] (ATCC® CRL-5820™), NCI-H2052 [H2052] (ATCC® CRL-5915™) and NCI-H2452 [H2452] (ATCC® CRL-5946™)
Authentication	The expression of cytokeratins 5/6 (CK5/6), pan-cytokeratin (AE1/AE3), and calretinin on these 3 cell lines were confirmed by Western Blot.
Mycoplasma contamination	All cell lines were tested negative for mycoplasma contamination
Commonly misidentified lines (See ICLAC register)	No misidentified cell lines.

Animals and other organisms

Policy information about studies involving animals; ARRIVE guidelines recommended for reporting animal research

Laboratory animals	Eight to ten-week-old female immunocompromised mice, NOD.CB17-Prkdcscid/J strain (NOD/SCID)
Wild animals	No wild animal were used.
Field-collected samples	NA
Ethics oversight	Animal Research Ethics Board at the Toronto General Research Institute (University of Toronto, Toronto, CA).

Note that full information on the approval of the study protocol must also be provided in the manuscript.

Clinical data

Policy information about clinical studies

All manuscripts should comply with the ICMJEguidelines for publication of clinical research and a completedCONSORT checklist must be included with all submissions.

Clinical trial registration	NCT00797719
Study protocol	The protocol was published with the results of the clinical trials in Lancet Oncol. 2021 Feb;22(2):190-197. doi: 10.1016/S1470-2045 (20)30606-9. Epub 2021 Jan 12. PMID: 33450184
Data collection	Recruitment was performed between Nov 1, 2008 and Oct 31, 2019. Data was collected by the principal investigator.
Outcomes	Survival was the endpoint. Survival was defined as time from diagnosis or surgery to death or last follow-up.

Flow Cytometry

Plots

Confirm that:

- [ ] The axis labels state the marker and fluorochrome used (e.g. CD4-FITC).
- [x] The axis scales are clearly visible. Include numbers along axes only for bottom left plot of group (a 'group' is an analysis of identical markers).
- [ ] All plots are contour plots with outliers or pseudocolor plots.
- [x] A numerical value for number of cells or percentage (with statistics) is provided.

Methodology

Sample preparation	CRL-5946 cells were detached and resuspended in FACs buffer. Cells were stained for 10 minutes at 4 oC with a CD16/CD32 Fc block (Biolegend Inc.) and then a combination of the following human-specific antibodies: GITRL-APC conjugated (Clone#109101, R&D Systems co.) and GITR-PE conjugated (Clone#621, BioLegend Inc.). All samples were then washed twice with FACS buffer and fixed with 2% Paraformaldehyde for at least 30 minutes. BD LSR II flow cytometer (BD Biosciences) and FlowJo V10 software (FlowJo LLC) was then used to analyze the expression of GITR and GITLR on MPM cells.
Instrument	. BD LSR II flow cytometer (BD Biosciences)
Software	FlowJo V10 software (FlowJo LLC)
Cell population abundance	At acquisition the entire population was the population of interest (100%).

Gating strategy	Only FSC/SSC gating was used. All negative and positive cells were shown on the graph.

☐ Tick this box to confirm that a figure exemplifying the gating strategy is provided in the Supplementary Information.
